# Geospatial Analysis of Accredited Lung Cancer Screening Facilities in Florida Reveals Suboptimal Alignment with High-Risk Populations

**DOI:** 10.1158/2767-9764.CRC-25-0535

**Published:** 2026-02-09

**Authors:** Salah-Eddin R. Komrokji, Luba Ayzenshtat, Haleh Sangi-Haghpeykar, Noman Ashraf, Sten H. Vermund, Abraham Schwarzberg, K. Eric Sommers, Etter Hoang, Eduardo M. Sotomayor, Matthew L. Anderson

**Affiliations:** 1 Tampa General Hospital Cancer Institute, Tampa, Florida.; 2 University of South Florida, Tampa, Florida.

## Abstract

Low-dose computed tomography (LDCT) plays a critical role in screening individuals for lung cancer. To better understand why its utilization remains poor, we analyzed the geospatial distribution of lung cancer screening programs in Florida to determine whether the distribution of these programs aligns with populations at greatest risk. Accredited programs offering LDCT were identified from public databases maintained by the American College of Radiology (ACR). Age-adjusted incidence and mortality for lung cancer were retrieved from the Florida Cancer Data System and Florida Department of Health. County-level demographic features were retrieved from the 2022 U.S. Census and Florida Department of Health. Univariate Pearson correlations and Kruskal–Wallis analyses revealed that screening programs in Florida are more likely to be located in counties with greater population density and a greater proportion of college-educated residents, physicians per capita, and residents with private insurance. In contrast, the number of facilities correlated inversely with the proportion of current smokers, White residents, and lung cancer incidence and mortality. In multivariate analyses, only smoking remained significantly associated with disease-specific incidence or mortality. No association between outcomes and the number of facilities in each county was identified. In conclusion, these findings indicate that the geographic distribution of certified screening programs in Florida does not align with lung cancer burden. Moving forward, strategies to address this mismatch may be helpful for improving the early detection of lung cancer.

**Significance:**

Our findings indicate that there is a significant misalignment between the distribution of ACR-accredited facilities offering LDCT for screening and the regions of Florida where the incidence and mortality of lung cancer are greatest. In contrast to previous reports examining national data, our data suggest that these geographic disparities limit the ability of populations most vulnerable to lung cancer to ACR-certified screening.

## Introduction

In 2024, an estimated 234,580 individuals were newly diagnosed with lung cancer in the United States (1). Despite a 23.4% decrease in its age-adjusted incidence between 1990 and 2019, lung cancer remains the second most common form of noncutaneous cancer (2). Lung cancers typically have a poor prognosis, with fewer than 29% of patients surviving more than 5 years (3). Because most are diagnosed at an advanced stage, lung cancer continues to account for nearly 20% of all US cancer deaths (3, 4).

Low-dose computed tomography (LDCT) is currently the only modality recommended by the U.S. Preventive Services Task Force (USPSTF) for population-based lung cancer screening in high-risk individuals (5, 6). To qualify for LDCT, current USPSTF guidelines require that asymptomatic individuals must be between 50 and 80 years of age, have at least a 20 pack-year smoking history, and currently smoke or have quit smoking within the past 15 years. LDCT helps to decrease mortality and improve survival largely because of its ability to detect lung cancers at earlier, more treatable stages. At least two randomized trials have reported that the use of LDCT reduces lung cancer mortality as much as 20% to 24% (7). These observations likely reflect the fact that individuals with screen-detected stage 1 lung cancer have been found to have an 88% 10-year survival rate compared with the 18.6% 5-year survival expected for all patients with lung cancer (8). A recent analysis of Behavioral Risk Factor Surveillance Survey (BRFSS) data found that 2.3% of survey participants between the age of 50 to 80 years were eligible for LDCT per 2021 USPSTF recommendations (9). However, multiple other studies have found that the uptake of LDCT remains disappointingly low, typically lower than 20% of the screen-eligible population (10–14). As a result, it is widely believed that efforts to improve screening uptake among eligible populations offer enormous potential to improve lung cancer survival.

Multiple sociodemographic factors, including insurance status, ethnicity, educational status, socioeconomic status, marital status, an established relationship with a primary care physician, and employment status, have all been previously shown to affect participation in lung cancer screening programs (15). Geographic factors may also contribute to low rates of screening (16). At least one recent study has reported that the incidence of lung cancer and its associated mortality are significantly higher in rural populations (17). This same study found that the incidence of lung cancer in the United States between 2013 and 2021 declined faster in urban populations than their rural counterparts. Other studies have found no differences in the rates at which rural and urban populations participate in lung cancer screening programs (18). Understanding these issues is complicated by the fact that ∼5% of the US population eligible for lung cancer screening resides more than 40 miles from the nearest screening facility (16). It currently remains unclear to what degree geographic factors limit participation in lung cancer screening and how best to address these issues at a regional or local level.

Florida is the third most populous state in the nation characterized by aging population for which racial, ethnic, income, and socioeconomic characteristics vary widely within its borders (19). It has also been previously shown to have both the greatest number of lung cancer screening facilities (*n* = 294; density = 5.3 per 100,000 at-risk persons) and among the highest proportions of population ≥65 years of age of any US state (with Maine and Vermont). A recent estimate suggests that only 2.4% of Florida’s high-risk population has been screened for lung cancer compared with the average national rate of 4.5% (20). We have undertaken the current study with the goal of determining whether the geographic distribution of accredited lung cancer screening facilities within Florida aligns with the geographic distribution of populations at greatest risk of developing and/or dying from this disease and how these relationships are potentially affected by demographic features.

## Materials and Methods

### Data collection and visualization

All data used for this study were retrieved from most up-to-date, publicly accessible sources available and retrieved on multiple dates in June 2024. Locations of lung cancer screening facilities registered with the American College of Radiology (ACR) were retrieved from ACR’s Lung Cancer Screening Locator Tool and verified against the GO2 database (19). Data documenting lung cancer–related demographics, such as incidence, mortality, and percentage of late-stage diagnoses, were obtained from the Florida Department of Health (20–22). Population and area for each Florida county were abstracted from the 2022 American Community Survey County (ACS) database. To calculate population density, the most recent population estimate for each county was divided by its respective area in square miles. Other data included the proportion of individuals aged >62 years, race/ethnicity, household median income, proportion of individuals with incomes below the federal poverty line, and proportion of individuals above the age of 25 years with only a high school or only a bachelor’s degree. The age threshold of 62 years was utilized because this age group had the least amount of missing data. Health funding status was defined as the proportion of individuals with different types of insurance, including Medicaid, Medicare, Veterans Administration, public health insurance private (commercially purchased or provided through an employer), and uninsured. Public health insurance was defined as Medicaid, Medicare, Veterans Insurance, state-funded programs, CHIP, and CHAMPVA, whereas private funding included options such as commercial and employer-based insurance. The number of physicians per capita analyzed in this study was obtained from the Florida Division of Medical Quality Assurance for Fiscal Year 2022 (23). No data were available about the sex/gender of subjects through these sources.

### Statistical analysis

#### Geospatial analysis

Utilizing the Google Geocoder API, addresses for each screening facility were converted into XY coordinates. The “summarize within” feature of ArcGIS (Esri, Inc., RRID: SCR_011081) was used to generate a table summarizing the number of facilities in each county. Univariate correlation matrices based on Pearson correlation coefficient were used to assess relationships between lung cancer incidence, mortality, and specific county-level demographic variables. Analyses that included population density were executed using the R Stats package (RRID: SCR_025968) to perform Spearman coefficient analyses because of the skewed nature of the population distribution across the state.

#### Descriptive/bivariate statistics

County-level data for incidence, mortality, and smoking were split into three levels using the Jenks natural breaks method (BAMMtools; RRID: SCR_027137). The Jenks natural breaks optimization method clusters data into K groups to reduce within-group variance and maximize the between-group variance (24). Using Jenks natural breaks, counties with lung cancer incidence less than 55.0 new lung cancers per 100,000 were categorized as “low”; 55.1 to 74.6 new lung cancers per 100,000 as “medium”; and greater than 74.6 new lung cancers per 100,000 as “high.” For mortality rates, counties with less than 40.8 deaths per 100,000 people were categorized as “low”; 40.8 to 68.4 deaths per 100,000 people as “medium”; and greater than 68.4 deaths per 100,000 people as “high.” Counties with current smoking rates less than 17.1% were categorized as “low”; 17.1% to 22.6% as “medium”; and greater than 32.4% as “high.” Kruskal–Wallis tests for skewed data were used to evaluate the association between the median number of lung cancer screening facilities per county among the binned groups for incidence, mortality, and current smokers. *Post hoc* Dunn tests with Bonferroni correction were performed to measure pairwise comparisons for each. Vargha and Delaney A (VDA) was used to estimate the effect size of *post hoc* tests using the R companion package.

### Multivariate analysis

To better understand the impact of lung cancer screening facilities on lung cancer burden, multiple multivariate regression models were utilized to assess the effects between relevant county-level demographic features and the geographic distribution of lung cancer burden in Florida (21). To address colinearity and potential interdependency between many of the individual demographic features being considered, variables were grouped into closely related, interdependent features identified in univariate analysis (e.g., payor status or race). Representative factors were selected based on the robustness of their correlation with other features in that group. Normality of the distributions was analyzed using the Shapiro–Wilk test. The individual features selected for inclusion for all models were selected based on their variance inflation factors (VIF), all of which were less than 5. Because of the nature of the publicly available data used, there was no missing data to manage in our analyses. A generalized linear model (GLM) with negative binomial distribution for the observed response (lung cancer screening program count) was used to estimate the association between lung cancer screening programs at a county level and a list of potential predictors. The response variable for the count of facilities per county was strongly skewed, frequently containing zeros and extreme outliers, leading us to explore Poisson or negative binomial models. Residual QQ plots for both Poisson and quasi-Poisson were not normally distributed, showing heteroscedasticity, along with both models being significantly overdispersed. A negative binomial model was selected over Poisson and quasi-Poisson models to account for overdispersion in the observed variance being greater than its mean. The negative binomial model had a ratio of deviance near 1, with normally distributed residuals, thus being our chosen model. A GLM with a gamma distribution and log link function was applied to estimate the difference in mortality attributed to lung cancer screening programs and various selected features. A gamma distribution with a log link was chosen because of the nature of the right-skewed distribution of mortality across the state, where the log link assumed a multiplicative or proportional effect. Finally, an ordinary least squares multivariate linear regression model was constructed to evaluate the individual effects of the variables on assessing lung cancer incidence. Residuals for both the gamma log link and ordinary least squares models were approximately normally distributed and homoscedastic.

Shapiro–Wilk normality test, Kruskal–Wallis test, Dunn test, linear model (LM), and GLM analyses were performed using the Stats package (RRID: SCR_025968). Negative binomial testing was conducted using the MASS package (RRID: SCR_019125). Overdispersion and residual testing were done using the DHARMa package (RRID: SCR_022136). Data transformations and graphical visualizations were executed using Tidyverse (RRID: SCR_019186). All other analyses and visualizations for this study unless otherwise specified were conducted using R Project for Statistical Computing (version 4.4.0, R Core Team, RRID: SCR_001905). Statistical significance was defined by two-sided *P* values less than 0.05. No randomization, blinding, group assignment, or size selection was performed as both the number of Florida counties and ACR-certified screening centers were fixed variables.

## Results

**Figure 1.**
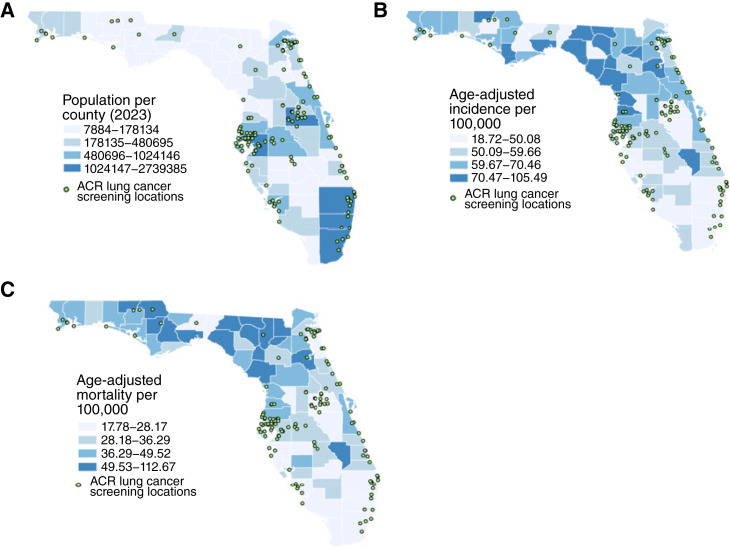
Maps of lung cancer screening facilities. **A,** Distribution of ACR-certified lung cancer screening facilities in Florida relative to 2022 ACS county-level population estimates. **B,** Distribution of screening facilities in Florida relative to the age-adjusted incidence of lung cancer per 100,000 individuals. **C,** Geographic distribution of lung cancer screening facilities in Florida mapped relative to age-adjusted mortality of lung cancer per 100,000 people.

**Figure 2.**
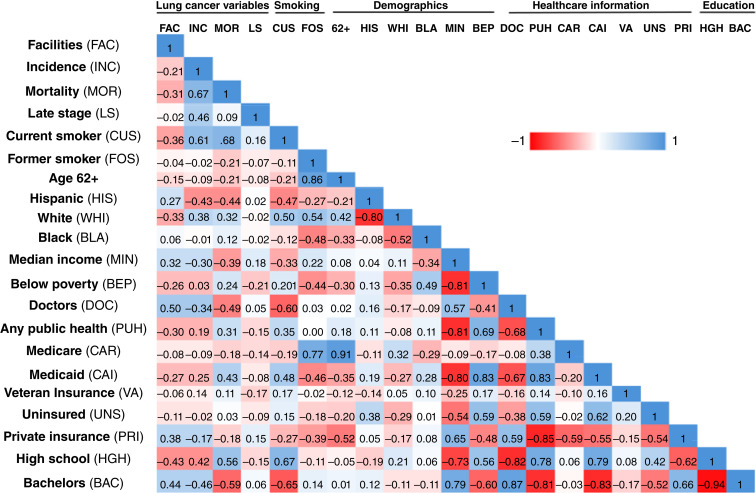
Correlation matrix for county-level demographic variables. Matrix of Pearson correlation coefficients estimated for county-level demographics.

We identified 141 ACR-recognized lung cancer screening centers in Florida, largely clustered around urban population centers in the state (Fig. 1A). When examined on a county level, we found that the number of facilities strongly correlated with its population density (r_s_ = 0.83, *P* ≤ 0.001) when statistical significance was defined by two-sided *P* values less than 0.05. However, both the incidence and mortality associated with lung cancer in Florida seemed to visually correlate inversely with the number of LDCT screening centers found in each county (Fig. 1B and C). Two-tailed Pearson correlation analyses confirmed an inverse correlation between the number of screening facilities and lung cancer mortality (r = −0.31, *P* = 0.01; Figs. 1B and 2). However, no relationship was observed between the county-level number of screening facilities and age-adjusted lung cancer incidence (r = −0.21, *P* = 0.10; Figs. 1C and 2).

To further assess relationships between lung cancer incidence, mortality, and demographic features, a broader two-tailed correlation matrix was assembled (Fig. 2). This revealed that the number of facilities in a county positively correlated with the number of higher physicians per capita (r = 0.50, *P* < 0.0005), proportion of college-educated residents (r = 0.44, *P* < 0.0005), the proportion of residents with private insurance (r = 0.38, *P* < 0.005), and median income (r = 0.32, *P* < 0.005). In contrast, the number of screening facilities correlated inversely with the proportion of residents with no more than a high school education (r = −0.43, *P* < 0.0005), its proportion of current smokers (r = −0.36, *P* < 0.005), White residents (r = −0.33, *P* < 0.005), age-adjusted mortality (r = −0.31, *P* < 0.05), proportion of Medicaid recipients (r = −0.27, *P* < 0.05), proportion of residents relying on public health insurance (r = −0.30, *P* < 0.05), and the proportion of individuals with household incomes below the federal poverty limit (r = −0.26, *P* < 0.05).

Further examination also uncovered multiple other previously identified associations (22). These include a strong correlation between county-level rates of current smoking with the proportion of residents with no more than a high school education (r = 0.67, *P* < 0.005), proportion of White residents (r = 0.50, *P* < 0.0005), Medicaid enrollees (r = 0.48, *P* < 0.0005), and the proportion of the population funded by any type of public health insurance (r = 0.35, *P* < 0.005). The proportion of current smokers in a county was found to correlate inversely (in decreasing order of robustness) with its proportion of residents with at least bachelor’s level education (r = −0.65, *P* < 0.005), doctors per capita (r = −0.60, *P* < 0.0005), proportion of Hispanic residents (r = −0.47, *P* < 0.0005), median income (r = −0.33, *P* < 0.005), and proportion of the population with private insurance (r = −0.271, *P* < 0.05).

We also examined the relationship between demographic variables, lung cancer outcomes, and the number of facilities per county (Fig. 2). As anticipated, we found that lung cancer incidence correlated robustly with the age-adjusted mortality of this disease (r = 0.67, *P* < 0.0005). We also found that lung cancer incidence correlated with the proportion of residents categorized as current smokers (r = 0.61, *P* < 0.0005), proportion of high school–educated residents (r = 0.42, *P* < 0.0005), late stage at diagnosis (r = 0.46, *P* < 0.0005), proportion of White residents (r = 0.38, *P* < 0.005), and the proportion of residents with Medicaid (r = 0.25, *P* < 0.05). In contrast, lung cancer incidence correlated inversely with the proportion of the population with at least bachelor’s level education (r = −0.46, *P* < 0.0005), Hispanic ethnicity (r = −0.43, *P* < 0.0005), and median income (r = −0.3, *P* < 0.05). Lung cancer–specific mortality was most robustly associated with the county-level proportion of current smokers (r = 0.68, *P* < 0.0005), proportion of residents with a high school education or less (r = 0.56, *P* < 0.0005), proportion receiving Medicaid (r = 0.43, *P* < 0.0005), proportion of White residents (r = 0.32, *P* < 0.005), and the proportion of residents with any form of public insurance (r = 0.31, *P* < 0.05). Mortality correlated inversely with the proportion of residents with at least a bachelor’s level education (r = −0.59, *P* < 0.0005), physicians per capita (r = −0.49, *P* < 0.0005), proportion of Hispanic residents (r = −0.44, *P* < 0.0005), and median income (r = −0.39, *P* < 0.005).

**Figure 3.**
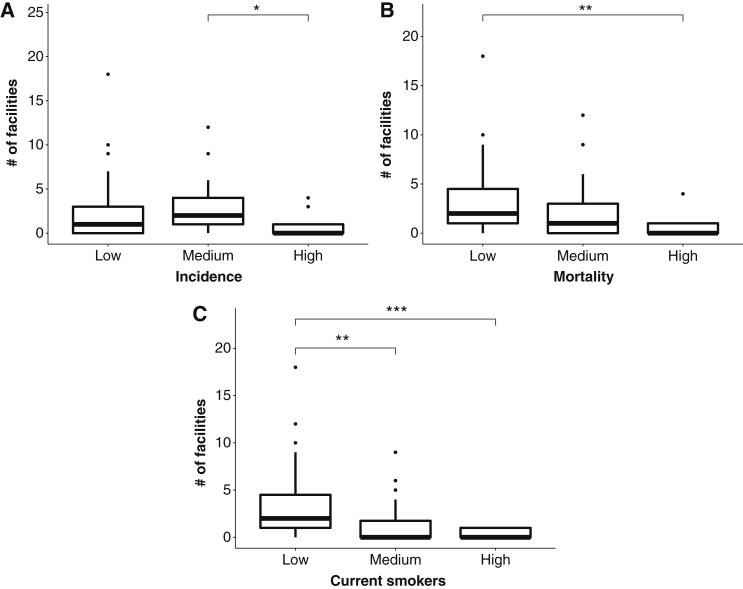
Kruskall–Wallis analyses evaluating relationship between county-level density of lung cancer screening facilities, proportion of current smokers, lung cancer incidence, and mortality. **A,** Number of screening facilities per county evaluated by ranked, binned, county-level age-adjusted incidence of lung cancer per 100,000. **B,** Comparison of lung cancer screening facilities per county by binned, ranked age-adjusted lung cancer mortality per 100,000. **C,** Number of lung cancer screening facilities per county by binned, ranked proportion of current smokers. *, *P* < 0.05; **, *P *< 0.01; ***, *P* < 0.005.

To confirm the results of univariate analyses reported above, we also used Kruskal–Wallis analyses to evaluate binned data. These analyses confirmed that associations between the median number of screening facilities, mortality, incidence, and proportion of current smokers were statistically significant [Fig. 3; H(2) = 10.01, *P* = 0.007; H(2) = 8.53, *P* = 0.01; and H(92) = 16,37, *P* = 0.0003, respectively]. Furthermore, *post hoc* Dunn tests indicated that the Florida counties with the lowest number of facilities had the highest lung cancer mortality [Fig. 3B; *P* = 0.005; confidence interval (CI), −31.6 to −4.3; VDA(B,A) = 0.78] and greatest proportion of current smokers [Fig. 3C; *P* = 0.0006; CI, −37.6 to −8.1; VDA(B,A) = 0.85]. Similar relationships were observed between the incidence and current smokers, such that counties with the highest lung cancer incidence had lower facilities than those with medium incidence [Fig. 3A; *P* = 0.01; CI, −31.3 to −2.6; VDA(B,A) = 0.76]; counties with the highest numbers of current smokers had lower screening facilities than those with medium numbers of current smokers [Fig. 1C; *P* = 0.009; CI, (3.0, 27.6); VDA(B,A) = 0.60].

In multivariate analysis, we found that the proportion of White residents (CE = −3.10; CI, −6.79 to 0.42; *P* = 0.04) was independently and inversely correlated with the number of facilities in each county whereas the number of physicians per capita (CE = 0.006; CI, 0.002−0.01; *P* = 0.00003) was positively and significantly correlated with the number of facilities (Table 1). For every one unit percent change in the proportion of White residents, the number of facilities decreased by 3.1%. No independent association between the proportion of current smokers and the number of facilities was observed (Table 1). We also found that the only variable which remained independently associated with the age-adjusted incidence of lung cancer was the proportion of current smokers (CE = 174.5; CI, 60.54−288.38; *P* = 0.003; Table 2). For every one unit change in incidence, the proportion of current smokers increased by 174.5%. Lung cancer mortality also remained independently associated with the proportion of current smokers (CE = 3.36; CI, 1.33−5.43; *P* = 0.002; Table 3). No independent association between the geographic density of lung cancer screening programs and either lung cancer incidence or mortality was observed. For every one unit change in mortality, the proportion of current smokers increases 3.36 fold.

**Table 1.** Multivariate analysis of county-level demographic features associated with number of facilities per county.

​	Coefficient estimate	SE	*z*-value	*P* value	95% CI
White	−3.10	1.52	−2.03	0.04	−6.79 to 0.42
Doctors	0.006	0.002	4.14	0.00003	0.002–0.01
Current smoker	4.45	5.56	0.80	0.42	−6.96 to 15.98
Medicaid	−7.88	6.16	−1.28	0.20	−22.52 to 6.21

**Table 2.** Multivariate analysis of selected county-level variables associated with lung cancer incidence.

​	Coefficient estimate	SE	t-value	*P* value	95% CI
LCS facilities	0.21	0.61	0.35	0.73	−1.01 to 1.44
Doctors	0.003	0.02	0.15	0.88	−0.04 to 0.05
White	16.28	18.06	0.90	0.37	−20.31 to 50.07
Current smokers	174.5	56.97	3.06	0.003	60.54–288.38
Medicaid	21.22	64.30	0.33	0.74	−107.35 to 149.79

Abbreviation: LCS, lung cancer screening.

**Table 3.** Multivariate analysis of selected county-level variables associated with lung cancer mortality.

​	Coefficient estimate	SE	t-value	*P* value	95% CI
LCS facilities	−0.003	0.01	−0.28	0.78	−0.02 to 0.02
Doctors	−0.00002	0.0003	−0.07	0.95	−0.0007 to 0.0007
White	0.50	0.33	1.49	0.14	−0.15 to 1.13
Current smokers	3.36	1.05	3.20	0.002	1.33–5.43
Medicaid	2.22	1.19	1.88	0.07	−0.12 to 4.57

Abbreviation: LCS, lung cancer screening.

## Discussion

Optimizing access to screening is critical for early detection and successful treatment of lung cancer. Here, we report that the geographic distribution of lung cancer screening facilities in the state of Florida does not align with the geographic distribution of its populations at greatest risk of developing or dying from this disease. Instead, lung cancer screening facilities are clustered geographically around regions with the highest population density. Practically speaking, this means that access to specialized lung cancer screening services tends to be in better educated counties with more physicians per capita and a lower proportion of residents who rely on public health insurance and are less likely to be current tobacco smokers—all demographic features potentially associated with a lower risk of developing lung cancer (25). It is readily conceivable that this misalignment contributes to low rates of lung cancer screening and high rates of lung cancer mortality in Florida by limiting the ability of established early detection programs to reach individuals at greatest risk (26).

As a starting point to decipher how demographic features are potentially associated with the access to LDCT screening programs, we examined the relationship between the number of facilities in each county and how the number of facilities and other variables relate to lung cancer burden. An interesting finding in our analysis is that the proportion of county residents relying on Medicaid had a robust and negative association with the number of lung cancer screening facilities. Multiple studies have previously reported that Medicaid increases the usage of lung cancer screening services as compared with even Medicare or private insurance (6, 23). The reason for the discrepancy between our results and those of prior studies is not immediately clear. However, this difference could be due to confounders such as education and rurality. The number of ACR-certified centers cannot be equated to actual usage of these centers and numbers of patients screened. Nonetheless, it is worth noting that Florida is one of 10 states that have not implemented Medicaid expansion fully under the Affordable Care Act; hence, many working Florida residents with low incomes are not covered by Medicaid. Interestingly, both univariate and multivariate analyses suggest that counties with a greater proportion of White residents tend to have fewer screening centers. This also contrasts with prior reports that examined nationwide data and reported that White individuals are more likely to participate in lung cancer screening than other racial and ethnic groups. This discrepancy may reflect the unique landscape of lung cancer screening in Florida (6, 23). It also suggests that further investigation to examine how rurality, drive times, and other, more granular evaluation of geographic features potentially affect rates of lung cancer screening may be useful for deciphering why the rate of lung cancer screening remains low and developing effective strategies to engage at-risk populations in screening.

Despite robust univariate correlations that fall along the lines of payor status and race/ethnicity, the only factor independently correlated with the geographic distribution of incidence or mortality of lung cancer in Florida in our multivariate analyses was the proportion of current smokers. Smoking is one of the most important risk factors for developing and dying from lung cancer (27, 28), yet the concentration of lung cancer screening programs on a county-level did not associate with the incidence or mortality of lung cancer in our state. At the outset of this project, we hypothesized that the number of screening facilities in a county might correlate positively with lung cancer incidence if ready access to screening led to larger numbers of patients being diagnosed with this disease. Similarly, the efficacy of LDCT as a screening strategy led us to assume *a priori* that lung cancer mortality in areas with a larger number of screening facilities would also be lower due to the LDCT’s demonstrated ability to diagnosis lung cancer at earlier, more curable stages. However, neither association was observed. There are many potential reasons that may explain this outcome. On one hand, it is possible that structural access alone is insufficient to overcome upstream behavioral and/or socioeconomic risk factors. However, it is important to note that the uptake of LDCT among high-risk individuals in Florida clinically eligible to receive these services remains low, perhaps linked to the inconvenient location of screening services for people at highest risk. Interestingly, the only factors that remain significantly associated in multivariate models with the county-level number of lung cancer screening centers are the number of physicians per capita and the proportion of White residents. This correlation potentially suggests that screening centers are located in areas in which care is more readily available. However, the inverse correlation with the proportion of White residents may indicate that screening centers are not being placed in the rural areas of Florida, which tend to be predominantly White. Moving forward, it will be important to consider each of these potential factors in greater depth.

Strengths of our study include the fact that it is one of the few studies to specifically examine geographic factors that potentially associate with low rates of lung cancer screening by analyzing the distribution of lung cancer screening centers in one of the most populous states in the United States. Importantly, all data used to conduct our analyses are publicly available, allowing for re-analysis and confirmation of our results. Thus, our observations may be valuable for helping in shaping public policy related to lung cancer screening and future access to screening and prevention services in a state with above average lung cancer mortality and lower rates of LDCT screening than average. Our results may be relevant to other US states although we have not yet begun to evaluate these questions specifically.

There are also several limitations inherent to our work. First, all data utilized relied on county-level demographics and measures. County-level data, although important, may not be sufficiently detailed to fully assess the impact of geographic features on lung cancer screening and may not be up to date, such as the last BRFSS survey. Moving forward, it will be important to evaluate functional geographic features, such as drive time, that more directly and functionally affect access to care. A second potential limitation to our analysis is the fact that the ACR registry may underreport the number of facilities providing LDCT screening services in Florida. The number of ACR-recognized lung cancer screening programs in Florida dropped from 294 in 2019 to 138 in 2023. This likely reflects the fact that the Centers for Medicaid and Medicare Services removed registry participation as a mandate for reimbursement in February 2022. Shortly thereafter, ACR officials noted a 25% decrease in the number registered programs (M. Simanowith, personal communication). Although no longer ACR certified, these centers may nonetheless continue to provide LDCT screening services to their communities. If so, their continued activity could confound the relationships observed in our analysis. Unfortunately, there are no publicly available databases which would allow us to directly address this potential pitfall. Additionally, this study cannot account for community outreach programs which can be impactful on participation in lung cancer screening programs. Although the results of the study may not be applicable to other states because of demographic and geographic differences, our methodologies can be applied to help assess geographic distribution in other states and nationwide.

It is important to note that the multivariate analyses conducted for this study were affected by clear relational issues, such as the interdependent nature of demographic features (e.g., payor status). As can be readily seen in Table 1, many of the demographic variables included in our analyses were collinear. This interdependency potentially obscures the significance and strength of correlations identified when mutually exclusive demographic features are simultaneously considered. Although we took several steps, such as assessing VIF scores and correctional analysis, and trialed multiple different technical approaches to our multivariate analysis to address these issues, we still found a high condition number in all our models accompanied by relatively weak r^2^ values. Furthermore, the effects of collinearity may have been amplified by the fact that there are only 67 counties in Florida. Nonetheless, we believe that the variables selected for the final analysis in Tables 1–3 represent a good proportion of demographics on a county level and provide useful insights on the nature of lung cancer screening facilities and how they potentially affect lung cancer burden in the state of Florida.

In conclusion, Florida is characterized by an uneven distribution of lung cancer screening centers. Counties that have the highest mortality rates and incidence of lung cancer tend to have the fewest lung cancer screening centers. Strategic planning involving public health organizations and hospitals, possibly for future satellite locations of lung cancer screening programs, could potentially reduce mortality in these areas. Other public health initiatives could help mobilize individuals in counties with no facilities to nearby ACR centers to increase the uptake of lung cancer screening or provide mobile screening vans that reach areas that may have increased difficulty accessing lung cancer screening.

## Data Availability

All data summarizing lung cancer–specific incidence and mortality utilized for this summary are publicly available through the Florida Cancer Data Service at https://fcds.med.miami.edu/inc/welcome.shtml. County-level smoking data were obtained from the Behavioral Risk Factor Surveillance System (25). County-level demographics, including health metrics and education levels, were abstracted from the U.S. Census Bureau and ACS 5-Year Estimates for 2022 (26).
